# Harnessing the power of endogenous pain control mechanisms for novel therapeutics: how might innovations in neuroimaging help?

**DOI:** 10.1097/SPC.0000000000000653

**Published:** 2023-06-22

**Authors:** Matthew A. Howard, Timothy Lawn, Olivia S. Kowalczyk

**Affiliations:** a Department of Neuroimaging, Institute of Psychiatry, Psychology and Neuroscience, King’s College London; b The Wellcome Centre for Human Neuroimaging, Institute of Neurology, University College London, London, UK

**Keywords:** descending modulation, fMRI, multimodal imaging, pain, spinal cord

## Abstract

This review explores the potential of using novel imaging approaches to deepen our understanding of descending modulatory mechanisms in pain, focussing on functional magnetic resonance imaging (fMRI) of the spinal cord and novel approaches to combining molecular and fMRI data. This review sheds light on the neural processes involved in pain modulation, paving the way for the development of targeted treatments.

**Recent findings:**

The reviewed literature demonstrates significant advancements in pain research. Recent studies show the potential of using fMRI to investigate the spinal cord’s role in pain modulation. Furthermore, novel analytical approaches integrating molecular and fMRI data show promise in elucidating the complex neurobiological processes underlying pain regulation. The main themes explored here include the identification of neurochemical markers associated with pain modulation and the characterisation of neural circuits involved in descending pain control.

**Summary:**

A comprehensive understanding of descending modulatory mechanisms in pain can inform the development of novel treatments, targeting dysfunction of these key pathways. By leveraging spinal fMRI and integrating molecular data into brain fMRI, researchers can identify potential therapeutic targets throughout the neuraxis. These advances may contribute to the development of personalised medicine approaches, allowing for tailored interventions based on individual pain profiles.

## INTRODUCTION

Chronic pain remains a major challenge for healthcare. Despite decades of effort and investment in the development of new treatments, patients continue to suffer, experiencing inescapable pain and poor quality of life ^1,2^. Current pharmacotherapies for chronic pain were largely originally developed for other therapeutic areas, at best offering modest relief and in only some patients ^3,4^. This necessitates the development of novel analgesic interventions, targeted to the patients who will respond. New, reliable readouts of pain mechanisms and pathophysiology are urgently needed ^5^.

Neuroimaging holds promise as an adjunctive technique for understanding pain mechanisms in health and disease ^6^. Combining neuroimaging readouts with machine learning has the potential to advance prediction of diagnostics, prognostics and treatment responses at the individual level, but this has yet to impact directly on patients seeking treatment ^7,8^. Here, we first briefly summarise developments in the understanding of the endogenous pain control system and how it might be exploited for novel pharmacotherapies. Next, we describe innovations in functional magnetic resonance imaging (fMRI) and how they may expedite and inform this particular analgesic development area ^9^. In particular, we focus on advances in two promising domains of mechanistic pain research: functional activity in the spinal cord (spinal fMRI); and attempts to link fMRI activity to neurotransmitter systems underlying pain and treatment responses. Finally, we consider the utility of multimodal approaches to understanding pain mechanisms and predicting treatment response, reflecting upon how these developments might add value.

**KEY POINTS**

- Endogenous pain control mechanisms are important in acute and chronic pain states and provide opportunities as targets for analgesia.
- Novel methods for pain reporting that inform underlying pathophysiology are necessary to complement existing self-report measures.
- Developments in functional MRI acquisition and analysis have promise in this endeavour, namely spinal fMRI and molecular-enhanced imaging.

## ENDOGENOUS PAIN CONTROL AND NOVEL AVENUES FOR PHARMACOTHERAPY

It was first recognised in the late 1970s that the brain and brainstem provide inhibitory/facilitatory control over incoming signals from the body, ultimately exerting their influence at the dorsal horn of the spinal cord. Since those early descriptions of diffuse noxious inhibitory controls (DNIC) ^10^, multiple descending control mechanisms have been identified that interact to influence moment-to-moment pain experiences, depending on other competing internal and external demands ^11▪▪^. Multiple neurotransmitter systems underpin these inhibitory/facilitatory influences, including gamma-aminobutyric acid (GABA), noradrenaline, serotonin and dopamine, in addition to opioidergic mechanisms ^12^, offering candidate mechanisms for therapeutic intervention. Crucially, these systems can become dysfunctional in chronic pain.

Both preclinical and clinical studies implicate monoaminergic descending control mechanisms in increased pain sensitivity and the maintenance of ongoing pain states ^12–14^. However, given the multiplicity of mechanisms through which top–down modulation is enacted, identifying which have become maladapted within a given patient is a key barrier to directing treatment. Psychophysical tests such as conditioned pain modulation (CPM; the so-called pain-inhibits-pain phenomenon) offer one way to assess the functionality of descending pain circuitry. Reports indicate that deficient CPM responses predict the likelihood of developing persistent post-surgical pain ^15^ and responses to drugs harnessing these mechanisms, including duloxetine (a serotonin and noradrenaline reuptake inhibitor) and tapentadol (a combined mu-opioid and noradrenaline reuptake inhibitor) ^16,17^. However, given the complexity and interrelatedness of these mechanisms, it is unlikely that one test will offer sufficient sensitivity and specificity for all patients. CPM is suggested to test the integrity of DNIC mechanisms only, so is unlikely to be informative of alterations in other descending control systems, such as those involved in stress-induced analgesia ^11▪▪^. Other psychophysical tests, including quantitative sensory testing (QST) offset analgesia or temporal summation of pain ^18–21^ offer varying levels of sensitivity to detect pathology in different pain phenotypes, but they are similarly unlikely to be sufficient in isolation, nor are they expressly intended to probe descending control mechanisms. Multimodal *convergent operations* approaches to understanding complex behaviour are not new ^22^ and recent reports indicate their potential in pain research. For instance, while no significant analgesic effect of duloxetine in painful knee osteoarthritis was observed on psychophysical, clinical or behavioural measures when considered in isolation, combining all measures predicted up to 70% of variability in treatment response ^23^. Crucially, it is exactly this variability that stymies progress in clinical trials and therapeutics development. A key challenge, then, is identifying predictors of treatment response – what is different mechanistically about the patients that do not respond to treatment and how can we obtain this information? We contend that new readouts informing the integrity of pain mechanisms in humans are needed. Ideally, these readouts should also back-translate. Here, we describe how key developments in neuroimaging may add value in this endeavour.

## IN-VIVO ASSESSMENT OF DESCENDING MODULATION IN THE SPINAL CORD USING FUNCTIONAL MAGNETIC RESONANCE IMAGING

As descending control mechanisms exert their control at the dorsal horn of the spinal cord, the ability to non-invasively observe these effects *in vivo* is highly desirable. Like the brain, the spinal cord demonstrates haemodynamic responses associated with neural activity, allowing it to be imaged in a similar manner using fMRI. To date, however, fMRI investigations in the spine remain comparatively sparse, largely due to inherent difficulties in acquiring sufficient quality data. The relatively small size of the cord, magnetic field inhomogeneity and the confounding effects of cardiac and respiratory cycles constitute major obstacles ^24^. However, continuous advances in acquisition and analytical methods (including static and dynamic magnetic field shimming and physiological noise correction ^25–28^ have made investigations of the brainstem and spinal cord tenable. Reports have described activity in accordance with the known anatomical organisation of the cord, demonstrating ipsilateral responses in the dorsal horn to noxious and somatosensory stimulation at vertebral levels corresponding to the body site. Similar accounts examining the role of the ventral horns underpinning the motor system exist (see Kinany *et al*. ^29▪▪^), but are outside the scope of this review. Similarly, resting-state fMRI reports have described synchronised oscillatory activity, known as functional connectivity (FC), between dorsal and ventral horns, both within and between hemicords and across vertebral levels ^28,30,31^. Modulation of spinal cord activity has also been described, with reductions in pain reports and fMRI responses following placebo administration ^32^ and during attentional analgesia, in which responses to evoked nociceptive heat stimuli are reduced when attention is directed away from pain by a distracting cognitive task ^33^. Imaging the brain and spine together is technically challenging, but connectivity relationships between the cord and periaqueductal grey, a core structure in descending pain control, have also been shown to predict subjectively reported pain scores following noxious heat stimulation ^34^. These studies, underpinned by recent reports parameterising the reliability of spinal fMRI ^28^,^29▪▪^, set the stage for examining the effects of descending pain control mechanisms in the cord and their modulation by pharmacological probes.

A recent such study has yielded important new insights, implicating the endogenous opioid system as a critical mechanism underpinning the downstream effects of attentional analgesia in the cord. In healthy volunteers, Oliva *et al*. ^35▪▪^ acquired fMRI data from the brain, brainstem and cervical cord simultaneously. They performed three attentional analgesia experiments: following administration of either naltrexone (an opioid antagonist), reboxetine (an noradrenaline reuptake inhibitor) or a placebo condition. As expected, subjectively reported pain decreased when participants were distracted by the task, but only following dosing with placebo or reboxetine; naltrexone abolished this effect. fMRI responses to varying intensities of thermal stimuli were identified in brain regions commonly associated with evoked pain experiences ^36^, but also in midbrain and brainstem descending pain control networks under known opioidergic (reviewed in Fields ^37^) and noradrenergic control ^11▪▪^. Spinal fMRI responses to differing heat intensities were reported at cervical levels C5/C6, corresponding to the appropriate dermatomal distributions of the forearm in the cord. Spinal responses also correlated with subjective pain reports. A proximal region in C6 demonstrated attentional analgesia, namely that distraction modulated responses to noxious heat, but only in the placebo and reboxetine conditions. Perhaps surprisingly, differential drug effects on attentional analgesia were not seen in brain or brainstem, but examining how brain-to-cord FC relationships were altered by the attentional task was informative. While both reboxetine and naltrexone weakened cortex to midbrain FC, naltrexone alone weakened connectivity between the brainstem and the spinal cord. These findings provide convergent behavioural and fMRI evidence that endogenous opioid systems mediate the effects of attentional analgesia. Further, this study demonstrates the potential of spinal fMRI to inform the development of new treatments exploiting descending pain control systems and beyond. Clearly many challenges remain. To date, almost all spinal fMRI reports are limited to investigating the cervical spine only, and have been largely performed in healthy volunteers. Further, despite considerable progress in acquisition and analysis methods, spinal data remain less sensitive to detecting subtle, spatially limited effects, compared to brain-only fMRI. However, we assert that as these nascent technologies mature, their translation into chronic pain patients might impact positively upon therapeutics development.

## MULTIMODAL COMBINATION OF MOLECULAR INFORMATION WITH FUNCTIONAL MAGNETIC RESONANCE IMAGING

While pharmacological imaging studies such as that described above are informative, a long-standing criticism is that BOLD, the physiological process underlying fMRI, is inherently agnostic to the involvement of neurotransmitter system(s) underpinning observed experimental effects. Studies using simultaneous fMRI and positron emission topography (PET), the latter being sensitive to particular neurotransmitters or receptor systems, exist, but remain few and far between. This is due to the many constraints imposed by PET, including restricted experimental designs, requiring radioactive tracers, invasiveness and significant cost. Thus, whilst offering key insights into the molecular underpinning of clinical pain and its modulation ^38^, novel scalable methods are needed to delineate modulatory neurotransmitter-related interventions and biomarkers for chronic pain.

An exciting solution to this problem has come in the form of novel analytic methods utilising relatively cheap and scalable fMRI data, enriching it with molecular information from other sources ^39▪▪^. The core premise of this approach is that fMRI responses are driven by biological processes underpinned by particular neurotransmitter systems. Accordingly, the pattern of activity seen across the brain should be at least partially explained by the distribution of the molecular machinery underlying that process. In practice, this means exploring the spatial similarity between fMRI data and the distribution of different molecular systems, largely group-averaged receptor density templates derived from publicly available PET data. Consider, for example, the effects of acute drug administration on neural responses; the resulting activity following dosing should roughly map onto the distribution of that drug’s primary receptors. An important early study demonstrated exactly that, with seven different drugs showing correlations between patterns of resulting regional cerebral blood flow (rCBF) and their respective pharmacological targets ^40^. Several recent investigations have extended this concept in neurological and psychiatric cohorts, importantly including clinical pain states. While arguably these combined multimodal techniques are best suited to discerning neurofunctional changes following a pharmacological challenge, Vamvakas *et al*. ^41^ recently demonstrated relationships between known neurotransmitter systems involved in generating chronic and post-surgical pain experiences and concomitant arterial spin labelling-derived changes in rCBF. These increases in rCBF observed during pain following third molar tooth extraction correlated positively with both mu-opioid and dopamine D_2_ receptor densities, and negatively with serotonergic 5-HT_2A_ receptor density. Similar but weaker relationships in opioid and dopaminergic receptor densities were also observed in patients with spontaneous pain secondary to osteoarthritis, but not with serotonin. This is expected, given the widely accepted view that living with chronic pain results in varying, individualised pathophysiological changes in peripheral and central nervous system structure and function ^42^. Importantly, this study demonstrates that fMRI readouts of real-world, clinically relevant pain experiences, both acute and chronic, can be methodologically linked to known neurotransmitter systems. These preliminary findings demonstrate the utility of this approach to identify novel pharmacological targets for pain relief.

Beyond simple spatial correlation analyses, additional work has considered more complex spatiotemporal relationships between receptor distributions from PET and fMRI data ^39▪▪^. Crucially, these offer exciting opportunities to generate novel biomarkers in chronic pain. In conventional FC analyses, interrelationships in the oscillatory activity between different regions of the brain are considered. By contrast, the recently developed REACT (Receptor Enhanced Analysis of Functional Connectivity by Targets) approach examines how temporal fluctuations in resting-state fMRI relate to the spatial arrangement of individual neurotransmitter systems ^43▪^. REACT is a powerful tool to disentangle receptor systems mediating the complex pharmacodynamics seen in acute drug studies ^44^, but also holds promise in being able to link pharmacoimaging through to treatment. Conventional resting-state fMRI studies do not offer clear opportunities for treatment, and knowing that a patient group shows differences in FC between certain brain regions does not offer an immediate opportunity for intervention. Molecular-enriched networks from REACT link this connectivity to the molecular systems upon which pharmacological treatments for pain act. Importantly, they allow us to exploit knowledge of the neurotransmitter systems underlying descending pain modulation to mechanistically target treatments to them.

A promising proof-of-concept study showed the utility of REACT to link aberrant connectivity related to several modulatory neurotransmitters through to prediction of treatment responses to a drug putatively acting upon them ^45▪▪^. Studying chronic painful knee osteoarthritis patients, the authors described receptor-enriched FC alterations relating to noradrenergic and serotonergic systems which are critical mediators of descending pain control. Baseline FC characteristics related to noradrenaline and serotonin (NET/SERT) transporters differentiated two discrete groups of knee osteoarthritis pain patients from matched pain-free controls. Excitingly, however, NET and SERT-enhanced FC characteristics predicted how osteoarthritis patients would respond to duloxetine prior to treatment. Further, they demonstrated that patients with dopamine transporter (DAT)-enhanced FC were more likely to respond to placebo treatment. Of note, no differences were observed in opioid-enriched FC in this study, suggesting that this facet of descending control is unaffected in these patient cohorts, or that any differences are not prominently represented in resting brain activity. This novel approach invites further, large scale data driven characterisation of molecular-network disruption within different chronic pain cohorts, which are readily utilisable as clear mechanistic links to the current and future pharmacopeia. Given the aforementioned challenges presented by variability in treatment responses across patients, predictive REACT-based biomarkers may prove critical moving forwards.

## THE FUTURE: MULTIMODAL ASSESSMENTS AND ADDED VALUE INSIGHTS FROM NEUROIMAGING

It is compelling to wonder what the result of behavioural tests such as CPM or a stress-induced analgesia, alongside a detailed somatosensory evaluation using QST, might have been in the investigations of osteoarthritis and duloxetine reported by Martins *et al*. ^45▪▪^. Similarly, what additional insights might have been derived from examining FC or responses to evoked stimulation in the brain and/or spine of individuals that did and did not respond to duloxetine in the study of Petersen *et al*. ^23^? We suggest that the broader inclusion of appropriately powered neuroimaging investigations is warranted, both in mechanistic studies and early phase trials seeking early signals of therapeutic efficacy. fMRI endpoints can of course be examined in isolation, and such investigations should provide unique and valuable insights regarding normative and perturbed functioning of pain mechanisms, adding value to conventional psychophysical, psychometric and clinical endpoints. However, it is clear that the formation of multimodal assessments as composite biomarkers ^46▪^ is the future for individualised precision healthcare ^47^. We suggest that the inclusion of fMRI endpoints in the construction of models that offer diagnostic, prognostic and treatment predictions will be of importance. Here we focussed on harnessing endogenous pain modulation mechanisms for novel therapeutics, but note that the underlying principles may be readily translated to other elements of the pain experience and more broadly to other neurological and psychiatric conditions. While we have uniquely considered pharmacotherapies, mechanistic evaluation of other therapies is also likely to be valuable ^48,49^. We do not suggest that the inclusion of imaging endpoints will be a panacea, but will provide vital adjunctive insights to the ‘gold-standard’ first person reports from our patients.
